# Peroxisome Proliferator-Activated Receptor α Agonist and Its Target Nanog Cooperate to Induce Pluripotency

**DOI:** 10.3390/jcm7120488

**Published:** 2018-11-27

**Authors:** Jungwoon Lee, Jinhyuk Lee, Yee Sook Cho

**Affiliations:** 1 Stem Cell Research Laboratory, Immunotherapy Convergence Research Center, Korea Research Institute of Bioscience and Biotechnology (KRIBB), Daejeon 34141, Korea; jwlee821@kribb.re.kr; 2 Genome Editing Research Center, Korea Research Institute of Bioscience and Biotechnology (KRIBB), Daejeon 34141, Korea; jinhyuk@kribb.re.kr; 3 Department of Biotechnology, KRIBB School, University of Science and Technology (UST), Daejeon 34113, Korea; 4 Department of Bioscience, KRIBB School, University of Science and Technology (UST), Daejeon 34113, Korea

**Keywords:** agonist, Nanog, peroxisome proliferator-activated receptor α, pluripotency, induced pluripotent stem cell, reprogramming

## Abstract

The pharmaceutical compounds that modulate pluripotent stem cell (PSC) identity and function are increasingly adopted to generate qualified PSCs and their derivatives, which have promising potential in regenerative medicine, in pursuit of more accuracy and safety and less cost. Here, we demonstrate the peroxisome proliferator-activated receptor α (PPARα) agonist as a novel enhancer of pluripotency acquisition and induced pluripotent stem cell (iPSC) generation. We found that PPARα agonist, examined and selected Food and Drug Administration (FDA) -approved compound libraries, increase the expression of pluripotency-associated genes, such as Nanog, Nr5A2, Oct4, and Rex1, during the reprogramming process and facilitate iPSC generation by enhancing their reprogramming efficiency. A reprogramming-promoting effect of PPARα occurred via the upregulation of Nanog, which is essential for the induction and maintenance of pluripotency. Through bioinformatic analysis, we identified putative peroxisome proliferator responsive elements (PPREs) located within the promoter region of the Nanog gene. We also determined that PPARα can activate Nanog transcription by specific binding to putative PPREs. Taken together, our findings suggest that PPARα is an important regulator of PSC pluripotency and reprogramming, and PPARα agonists can be used to improve PSC technology and regenerative medicine.

## 1. Introduction

Pluripotent stem cells (PSCs) have unique properties of unlimited self-renewal and pluripotency to differentiate into all kinds of cell types in the body, which represent valuable biomaterial for applications in regenerative medicine [1]. The pluripotency of PSCs is tightly controlled by a network of core transcriptional regulatory factors, such as Oct4, Sox2, and Nanog [2,3], as well as nuclear receptors and ligand-dependent transcription factors, such as Esrrb, the Nr5A family, and Nr0b1 [4,5]. Various combinations of these transcription factors have been tested and identified for somatic cellular reprogramming to pluripotency, particularly the four “Yamanaka factors (Oct4, Sox2, Klf4, and c-Myc, abbreviated as OSKM)” [6,7] and other alternative factors (Oct4, Sox2, Nanog, and Lin28) [8]. Alternative transcription factors that can replace classical reprogramming factors or enhance reprogramming efficiency have been suggested [9,10]. Esrrb can replace Klf4 [11] or Sox2 [12], and Nr5A2 can replace Oct4 [13] in the derivation of induced pluripotent stem cells (iPSCs). Nr0b1 and Nanog can stabilize induced pluripotency synergistically during somatic cellular reprogramming [14]. In addition to transcription factors, small molecule compounds, offering ease of use and administration and cost-effectiveness, have been extensively tested, and the use of pre-existing small molecules has been suggested to advance iPSC generation and utility [15,16].

The peroxisome proliferator-activated receptors (PPARs) are ligand-dependent transcription factors that belong to a superfamily of nuclear hormone receptors [17,18] and play critical roles in numerous cellular processes, including lipid metabolism, inflammatory pathway, glucose homeostasis, cell cycle control, differentiation, development, and extracellular matrix remodeling [17,18,19,20]. PPARs share a highly conserved structure and molecular mode of action as a heterodimer with the retinoid x receptor (RXR), recognizing specific DNA sequences in target genes known as peroxisome proliferator response elements (PPREs) [21,22,23]. PPREs are characterized by a direct repeat sequence of the consensus hexanucleotide AGGTCA interspaced by a single nucleotide [23,24]. Each PPAR isoform regulates different target genes, thereby modulating distinct biological processes [24]. However, the mechanism underlying the specificity of regulation and the degree of acceptable sequence variability in PPREs is unclear [24,25]. In addition, functional PPREs have been identified only in a limited number of target genes, although numerous genes are known to be regulated by PPARs [25,26].

PPARs comprise the three isoforms, PPARα, PPARγ, and PPARβ/δ, which are differentially expressed in several tissues: PPARα and PPARβ/δ are expressed ubiquitously, whereas PPARγ is mainly expressed in macrophages, adipocytes, and colon cells [17,18]. During *Xenopus laevis* Daudin gastrulation, the pluripotency-related chromatin signature (H3K27me3) can be recognized by PPARβ [27]. PPARγ agonists have been reported to induce the loss of leukemia inhibitory factor (LIF)-dependent self-renewal of mouse embryonic stem cells (ESCs) and adipocyte differentiation [4,28,29]. In combination with the Rho-associated kinase (ROCK) inhibitor Y-27632, a PPARγ agonist blocked apoptosis and enhanced the cloning efficiency of human PSCs after dissociation [30]. However, the roles of PPARα, which is the best characterized isoform, remain not fully understood in PSCs.

Here, we demonstrate that PPARα agonists, such as fenofibrate, have a positive effect on the generation of iPSCs by a high-throughput screening strategy from selected FDA-approved compound libraries using genetically homogeneous secondary mouse embryonic fibroblasts (MEFs) harboring doxycycline (dox)-inducible OSKM transgenes. The PPARα-mediated enhancement of reprogramming efficiency was found to be associated with PPARα-mediated Nanog promoter activation. Taken together, our findings may provide new roles for the PPARα agonist as a PSC fate controller in somatic cellular reprogramming and iPSC technology.

## 2. Experimental Section

### 2.1. Chemicals and Reagents

Fenofibrate (#4113), WY14643 (#1312), and A769662 (#3336) were purchased from Tocris (Tocris, Minneapolis, MN, USA) and dissolved in dimethyl sulfoxide (DMSO; Sigma-Aldrich, St. Louis, MO, USA). Cell culture medium (DMEM-high glucose), fetal bovine serum (FBS), glutamine, nonessential amino acid (NEAA), penicillin-streptomycin, and trypsin/EDTA were acquired from Thermo Fisher (Thermo Fisher Scientific, Waltham, MA, USA).

### 2.2. Mice

C57BL/6J and R26^rtTA^; Col1a1^4F2A^ and BALB/c-nude mice were obtained from Jackson Laboratory (Bar Harbor, ME, USA) and maintained at Korea Research Institute of Bioscience & Biotechnology (KRIBB). All mice maintenance and experiments were approved by the Institutional Animal Care and Use Committee of KRIBB.

### 2.3. Cell Culture

For the feeder-dependent condition, undifferentiated mouse PSCs were cultured on gamma-irradiated MEF feeder layer in PSC medium composed of PSC basal medium DMEM-high glucose supplemented with 2 mM glutamine, 1% NEAA, 0.1 mM β-mercaptoethanol (Sigma-Aldrich, St. Louis, MO, USA), 100 units/mL penicillin, 100 μg/mL streptomycin, 1% NEAA, 15% FBS, and 1000 U/mL leukemia inhibitory factor (LIF) (Millipore, Billerica, MA, USA). In the feeder-free condition, the cells were maintained on 0.2% gelatin-coated dish in PSC medium. The PSC medium change was performed every other day. In 2–3 days, cells were washed once with PBS and treated with 0.25% trypsin/ Ethylene Diamine Tetraacetic Acid (EDTA) and dissociated into a cell and then transferred to 0.2% gelatin-coated dish.

### 2.4. Somatic Cellular Reprogramming

Following the protocol, 4F2A MEFs were isolated from embryonic day 13.5 (E13.5) embryos of the single Dox inducible transgenic mouse strains that express four reprogramming genes OSKM separated by three sequences encoding 2A self-cleaving peptides from the Col1a1 locus [31]. Briefly, internal organs and heads of embryos were removed before MEFs isolation, and then MEFs were expanded in DMEM-high glucose supplemented with 10% FBS, 100 units/mL penicillin, 100 μg/mL streptomycin, and 1% NEAA. The reprogramming scheme is in accordance with the published procedures [31,32].

### 2.5. Alkaline Phosphatase (AP) Staining

Alkaline Phosphatase (AP) staining was performed using a commercially available AP detection kit (Sigma-Aldrich, St. Louis, MO, USA), as previously reported [32]. Briefly, cells were fixed by fixation solution composed of citrate solution, acetone, and 37% formaldehyde for 30 s at room temperature. Then cells were stained with AP staining solution in the dark. Staining solution was removed and washed with distilled water. AP stained colonies were observed by light microscopy.

### 2.6. Immunocytochemistry

Cells were fixed with 4% paraformaldehyde for 10 min at room temperature. Then cells were permeablized with 0.1% Triton X-100 for 30 min, and washed with Phosphate buffered saline with Tween-20 (PBST) followed by blocking was performed by using 4% bovine serum albumin (BSA). Primary antibodies in blocking buffer were treated and then incubated overnight at 4 °C. Alexa Fluor 594 and Alexa Fluor 488 (Life Technologies, Carlsbad, CA, USA) were used for secondary antibodies. The primary antibodies used for PSCs were anti-Oct4 (sc-5279, Santa Cruz, Dallas, TX, USA), -Nanog (ab80892, Abcam, Cambridge, UK), and -SSEA1 (sc-21702, Santa Cruz, CA, USA).

### 2.7. Three Germ Layer Differentiation in Vivo and in Vitro

For in vitro three germ layer differentiation, cells were trypsinized and transferred to petri dishes in the PSC medium without a LIF. After a week, aggregated cells were plated onto 0.2% gelatin-coated Lab-Tek 4 well chamber and cultured for another week. Cells were stained with anti-Tuj1 (PRB-435P, Covance, Princeton, NJ, USA), -Nestin (sc-23927, Santa Cruz, CA, USA), -Desmin (AB907, Millipore, Burlington, MA, USA), -α-SMA (A5228, Sigma Aldrich, St. Louis, MO, USA), -Foxa2 (07-633, Millipore, Burlington, MA, USA), and -Sox17 (sc-17355, Santa Cruz, CA, USA). Teratoma formation was performed for in vivo three germ layer differentiation. iPSCs were harvested in single cell using 0.25% trypsin/EDTA. Cells were injected with Matrigel by subcutaneous injection on 4-week-old BALB/c-nude mice. Two weeks after injection, teratomas were isolated from mice and fixed overnight in 4% paraformaldehyde at 4 ℃ and then embedded in paraffin. Paraffin-embedded tissues were sectioned, stained with hematoxylin and eosin, and were observed using light microscopy to examine whether all three germ layer tissues were presented.

### 2.8. Quantitative Real-Time PCR

Total RNA isolated using a Rneasy Kit (Qiagen, Valencia, CA, USA) was reverse transcribed using a First Strand Synthesis kit (Invitrogen, Carlsbad, CA, USA). Quantitative real-time RT-PCR analysis was performed in triplicate using 1/50 of the reverse transcription reaction in an ABI Prism 7500 (Applied Biosystems, Foster City, CA, USA) with QuantiTect SYBR green PCR kit (Qiagen, Venlo, The Netherlands). The following primers were used for qPCR: Oct4, Forward 5′-CTTCACCACACTCTACTC; Reverse 5′-CCAGGTTCTCTTGTCTAC; Nr0b1, Forward 5′-TCCAGGCCATCAAGAGTTTC; Reverse 5′-ATCTGCTGGGTTCTCCACTG; Nanog, Forward 5′-TGAGCTATAAGCAGGTTAAGAC; Reverse 5′-CAATGGATGCTGGGATAC TC; Nr5a2, Forward 5′-AGATGCCAGAAAACATGCAA; Reverse 5′-TATCGCCACACA CAGGACAT; PPARα, Forward 5′-TATTCGGCTGAAGCTGGTGTAC; Reverse 5′-CTGGCATTTGTTCCGGTTCT; Rex1, Forward 5′-GATCCGCAAACACCTGCTTT; Reverse 5′-CCAAGTGTTG TCCCCAAATACC. The quantitation of the relative expression levels of the marker genes was achieved by normalizing for the endogenous glyceraldehyde-3-phosphate dehydrogenase (GAPDH) using the delta CT method, as described previously [32].

### 2.9. Reporter Gene Assay

The plasmid pSG5-PPARα and Nanog5P [33] were a gift from Bruce Spiegelman (Addgene plasmid # 22751) and Austin Cooney (Addgene plasmid # 16337), respectively. pGL3-basic promoter luciferase reporter (Promega, Madison, WI, USA) and pSG5 null vector (Agilent, Santa Clara, CA, USA) were used as controls. Renilla luciferase activities were used to normalize transfection efficiencies. Cells were transfected using Lipofectamine 2000 (Thermo Fisher Scientific, Waltham, MA, USA). 48 h after transfection, luciferase activity was measured using Dual-Luciferase Reporter Assay System according to the manufacture’s protocol (Promega, Madison, WI, USA). Briefly, cells were lysed with passive lysis buffer for 15 min on a gently shaking orbital shaker. The luminescence of the samples was measured following the manufacturer’s assay protocol for 96-well plates.

### 2.10. Bioinformatic Analysis and Chromatin Immunoprecipitation

Putative PPARα/RXR or PPARα consensus sequence identification was performed by using JASPAR and PROMO (version 8.3 of TRANSFAC) databases. Chromatin immunoprecipitation (ChIP) assay was performed by Pierce Agarose ChIP Kit (Thermo Fisher Scientific, Waltham, MA, USA) following the manufacturer’s instructions. Cells were crosslinked with 1% formaldehyde, washed with cold PBS, and the cells were lysed. The genomic DNA was sonicated, immunoprecipitated with an anti-PPARα antibody (sc-9000, Santa cruz, CA, USA), and then analyzed by qPCR using specific primers able to amplify the regions including putative PPARα/RXRα and PPARα consensus sequence (Forward 5′-GTGAGTTGGAAGCCAG; Reverse 5′-CCTCAGCCGTCTAAGC).

### 2.11. Statistical Analysis

Results are shown as the mean + standard error of the mean (SEM) (*n* = 3). The statistical significance was determined using a Kruskal–Wallis test with Dunn´s multiple comparison (* *p* < 0.05, ** *p* < 0.01, *** *p* < 0.001).

## 3. Results

### 3.1. Experimental Results

#### 3.1.1. PPARα Agonists Enhance Cellular Reprogramming to Derive iPSCs

**Figure 1:**
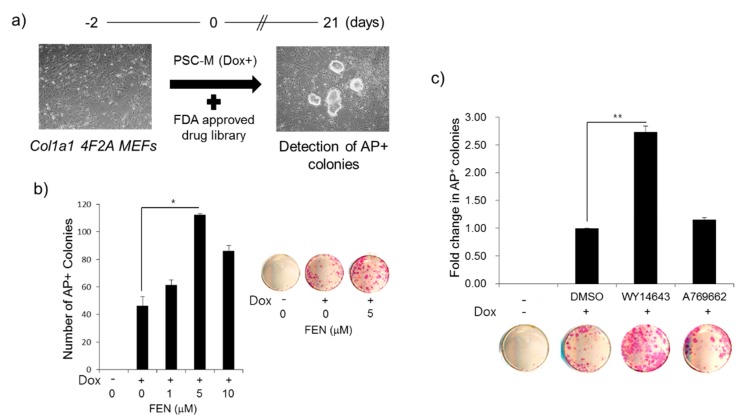
Screening and identification of PPARα agonist fenofibrate (FEN) from selected FDA-approved compound libraries that promote mouse embryonic fibroblasts (MEFs) reprogramming. (**a**) Schematic experimental procedure. The Col1a1 4F2A MEFs were seeded at 7.5 × 10^3^ cells per well in 96-well plates. After two days, cells were treated with or without compound and cultured in Dox to activate OSKM expression. The number of AP+ colonies on day 21 post-treatment was used as a measure of the reprogramming efficiency. (**b**) Dose-dependent enhancement of reprogramming efficiency by FEN. OSKM-induced cells were incubated with or without FEN at the indicated concentrations. Representative images of AP+ colonies per well were presented in the right panel. (**c**) The enhancement of cellular reprogramming efficiency by selective PPARα agonist WY14643 but not AMPK activator A769662. The Col1a1 4F2A MEFs were seeded, and incubated with or without drug in pluripotent stem cells (PSC) medium supplemented with or without Dox. The number of AP+ colonies on day 21 post-treatment was used as a measure of the reprogramming efficiency. Representative images of AP+ colonies per well were presented in the bottom panel.

To facilitate the cellular reprogramming process using pre-existing small molecule compounds, we conducted a high-throughput chemical screen of the collection comprised of 665 FDA-approved compounds (Table S1) using *Col1a1 4F2A* MEFs [31]. The reprogramming procedure is in accordance with published procedures [31,32], with modifications (Figure 1a). *Col1a1 4F2A* MEFs can generate iPSCs in PSC medium by Dox, with or without drug (Figure 1a), as previously reported [31]. We evaluated cellular reprogramming efficiency by counting the number of alkaline phosphatase (AP)-positive ESC-like colonies at 21 days of Dox induction. Among the tested FDA-approved compounds, the PPARα agonist, third generation fibrate, fenofibrate (FEN) was found to be the most effective compound in the generation of AP-positive ESC-like colonies. FEN improved the reprogramming efficiency in a dose-dependent manner, and the maximal effect of FEN on improving reprogramming efficiency was 5 μM (~2.42-fold) (Figure 1b). We randomly isolated two iPSC colonies derived by FEN treatment (FEN-iPSCs) and verified their pluripotency by immunostaining for pluripotency markers, such as Oct4, Nanog, and SSEA1 (Figure S1a), and in vitro and in vivo three-germ layer differentiation (Figure S1c,d). We also confirmed the normal karyotype of FEN-iPSCs (Figure S1b).

FEN is well known as a typical PPARα agonist, as well as an AMP-activated protein kinase (AMPK) activator in an independent manner of PPAR receptor activation [34,35,36]. To clarify the effect of FEN during the reprogramming process, we tested whether the selective PPARα agonist WY14643 and the AMPK activator A769662 have a simulative effect on iPSC generation. Distinctively, WY14643 improved the reprogramming efficiency (~3-fold) compared to the control DMSO treatment, but A769662 had no noticeable effect (Figure 1c). These results suggest that PPARα agonists are able to accelerate somatic cellular reprogramming.

#### 3.1.2. FEN-Stimulated PPARα during the Early Stage of Reprogramming Contributes to the Improved Reprogramming Efficiency

**Figure 2:**
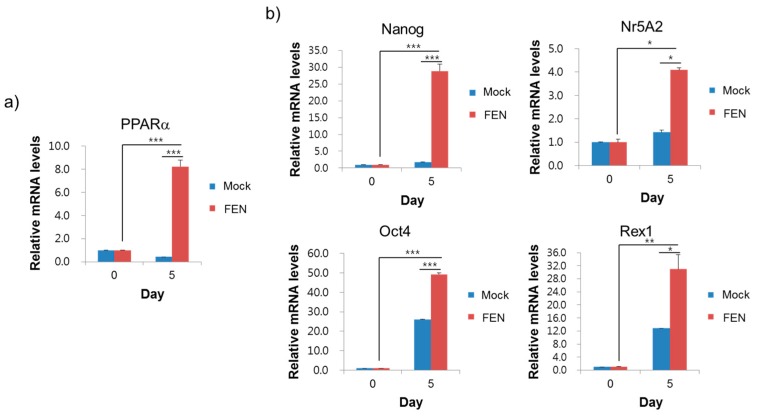
FEN significantly increases PPARα and pluripotency-associated genes. (**a**) Expression of PPARα in OSKM-induced cells with or without FEN 5 μM for 5 days. The results were normalized to GAPDH and expressed as a fold-increase over Mock (DMSO, vehicle control). (**b**) Expression of the pluripotency-associated genes (Nanog, Nr5A2, Oct4, and Rex1) in OSKM-induced cells with or without FEN 5 μM for 5 days. The results were normalized to GAPDH and expressed as a fold-increase over Mock (DMSO, vehicle control).

PPARα agonists have been demonstrated to increase the expression of PPARα [37,38,39]. With FEN treatment, PPARα expression was significantly increased in the early stage of reprogramming at day 5 (Figure 2a and Figure S2b), but other PPARs, such as PPARγ, were not (data not shown). Additionally, in response to FEN treatment, the expression of the pluripotency-associated Nanog (~16.5-fold), Nr5A2 (~1.9-fold), Oct4 (~2.4-fold), and Rex1 (~2.9-fold) genes was significantly increased in reprogrammed cells that were in an early stage of reprogramming (day 5) compared to the control DMSO treatment (Figure 2b). Likewise, the protein levels of PPARα, Nanog, and Oct4 were significantly increased in response to FEN treatment compared to the control (Figure S2b). Noticeably, Nanog expression was most upregulated by FEN treatment during the same time period (Figure 2b). The endogenous expression level of the PPARα gene was not significantly different between PSCs, such as fully reprogrammed iPSCs and undifferentiated ESCs, and nonpluripotent MEFs (Figure S2a). These results indicate that the PPARα-mediated beneficial effect on cellular reprogramming may be limited by pluripotency reestablishment rather than maintenance and this effect is dependent on the FEN-stimulated PPARα-mediated regulation of genes, particularly pluripotency-associated genes, such as Nanog, Nr5A2, Oct4, and Rex1.

#### 3.1.3. Prediction of Putative PPARα Binding Elements in the Nanog Promoter Region

**Figure 3:**
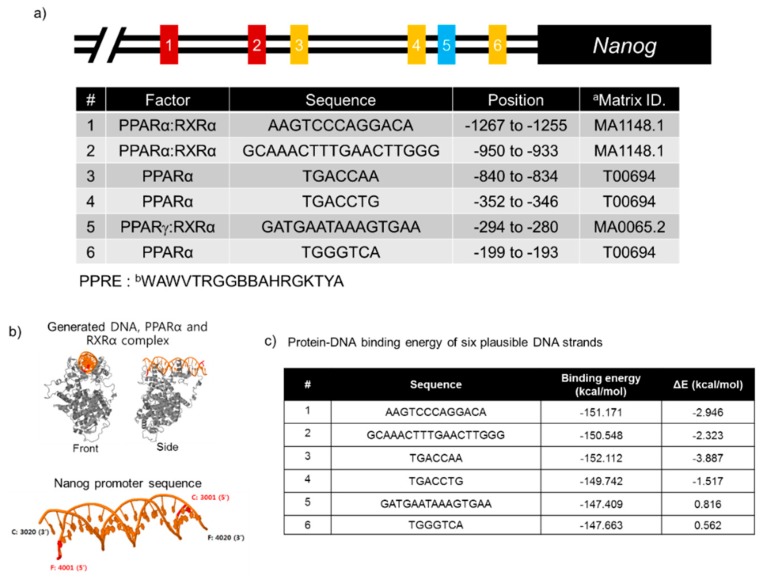
Predictive peroxisome proliferator response elements (PPREs) in the Nanog promoter region. (**a**) Putative PPARα/RXRα, PPARα, or PPAR/RXRα binding sites within −1.5 kb of the transcription start site of the Nanog promoter were identified by using JASPAR and PROMO (version 8.3 of TRANSFAC). a. MA1148.1 and MA0065.2 are the matrix IDs for JASPAR, and T00694 is the matrix ID for PROMO. b. W: A or T, V: A or G or C, R: A or G, B: C or G or T, K: G or T, and Y: C or T. (**b**) Structure construction of the mouse PPARα/RXRa and Nanog DNA complexes, which are drawn by gray and yellow cartoons, respectively. (**c**) Calculation of protein-DNA binding energy. The protein-DNA binding energies of six plausible DNA strands are numbered from 1 and 6 in the first column. The ‘Seq’ column represents the corresponding sequences with the predicted or revealed DNA sequences as shown (**a**). ‘Binding energy’ indicates the protein-DNA binding energy in units of kcal/mol. ‘ΔE’ is the relative binding energy with respect to the reference energy, which is the average energy of all binding energies on the whole sequence.

PPARα plays an important role in lipid and lipoprotein metabolism as a master regulator of fatty acid oxidation (FAO) [24] and shows numerous protective effects, including anti-inflammatory, antioxidant, metabolic control, and apoptotic regulation [40,41,42]. Nevertheless, limited numbers of PPARα-regulated genes harboring functional PPREs have been determined. Nanog is well known to be involved in the control of PSC self-renewal, naïve pluripotency, tumor initiation, and the chemoresistance of tumor-initiating stem-like cells (TICs) [43,44]. In addition, Nanog ChIP-seq analysis showed that oxidative phosphorylation (OxPhos) and FAO were involved in Nanog-mediated oncogenic pathways [43]. To explore the possible novel PPARα target in the control of pluripotency and reprogramming, we speculated and confirmed whether functional PPREs could be occupied by PPARα in the Nanog promoter. Significantly, we identified the putative PPARα/RXRα or PPARα binding sites within −1.5 kb of the transcription start site of Nanog by using JASPAR and PROMO (version 8.3 of TRANSFAC). Six putative PPREs within the Nanog promoter, including consensus sequences for PPARα/RXRα, PPARα, or PPARγ/RXRα, were selected (Figure 3a) to measure binding energies between the DNA strand and the PPARα/RXRα complex. The computational calculation of protein-DNA binding energies requires a three-dimensional structure. Thus, homology modeling to generate the mouse PPARα/RXRα complex with the DNA binding region was performed by PQR-SA (Pseudo Quadratic Restraints with Simulated Annealing) [45], which can find several template structures in the PDB using HHblits [46]. The calculated structures of mouse PPARα and RXRα (Figure 3b) and their validation metrics were generated in terms of radius of gyration, radar charts on various protein quality scores, and distance heat map (Figure S3a,b). The template structures for mouse PPARα and RXRα were obtained from human PPARγ (PDB entry code: 3DZY) and RXRα (PDB entry code: 1K7L), and the similarities of each of the two structures were 3.4 Å and 3.6 Å, respectively, indicating that the homology structures were similar to each other, although the ligand/DNA binding domains were slightly different (Figure S3a,b).

All putative sequences with extended 20 bp nucleotides were structured and moved by a base on the template structure of the PPARα/RXRα complex, which comprised two chains (C and F) sharing the backbone structure (phosphate and sugar rings) (Figure 3b). The DNA structure was generated using the CHARMM (Chemistry at HARvard Macromolecular Mechanics) modeling program [47]. The combined structure (DNA strand, PPARα and RXRα) was energy minimized on an implicit solvation model (GBSW: Generalized Born model with SWitch function) [48] and simulated with all heavy atoms fixing to prevent the system from exploding because of the many clashes caused by superimposition. The protein-DNA interaction energies on all of the combined structures were measured by subtracting protein only (E_prot_) and DNA only energies (E_DNA_) from the total energy of the system (E_total_). The binding energy and relative binding energies (ΔE) on six plausible DNA strands were estimated by the CHARMM program, resulting in three regions, AAGTCCCAGGACA (−1267 to −1255), GCAAACTTTGAACTTGGG (−950 to −933), and TGACCAA (−840 to −834), from the transcription start site of Nanog that showed a greater than 2-fold increase in ΔE compared to reference elements (Figure 3c). These results suggest that at least three PPARα binding elements exist within the proximal Nanog promoter.

#### 3.1.4. FEN Upregulates the Nanog Promoter via the PPARα Regulatory Pathway

**Figure 4:**
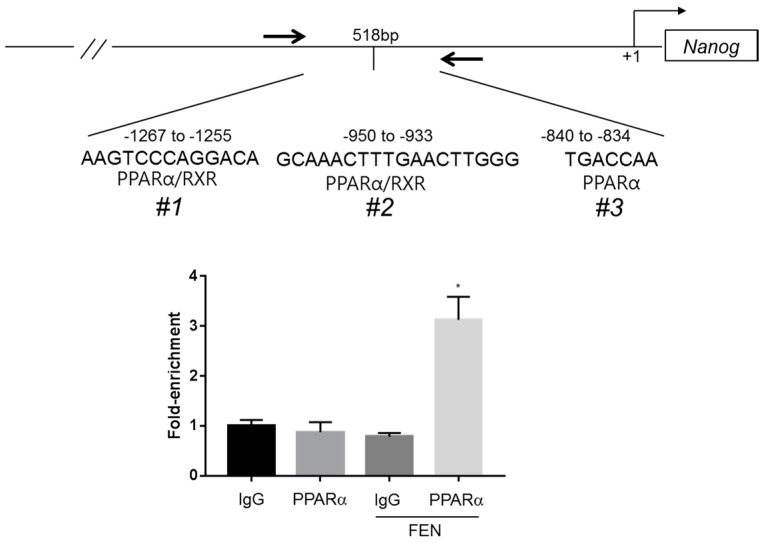
PPARα directly binds the Nanog promoter. ChIP assay demonstrates that PPARα binds to the regions including putative PPRE motifs at −1267 to −1255, −950 to −933, and −840 to −834 bp upstream of the Nanog promoter in vivo. Chromatin samples were prepared from OSKM-induced cells at 5 days after treatment with or without FEN and immunoprecipitated with antibodies against rabbit IgG or PPARα. qPCR detection was performed using specific primers for regions of 518 bp that included putative PPARα binding sites as shown in the schematic in the upper panel.

**Figure 5:**
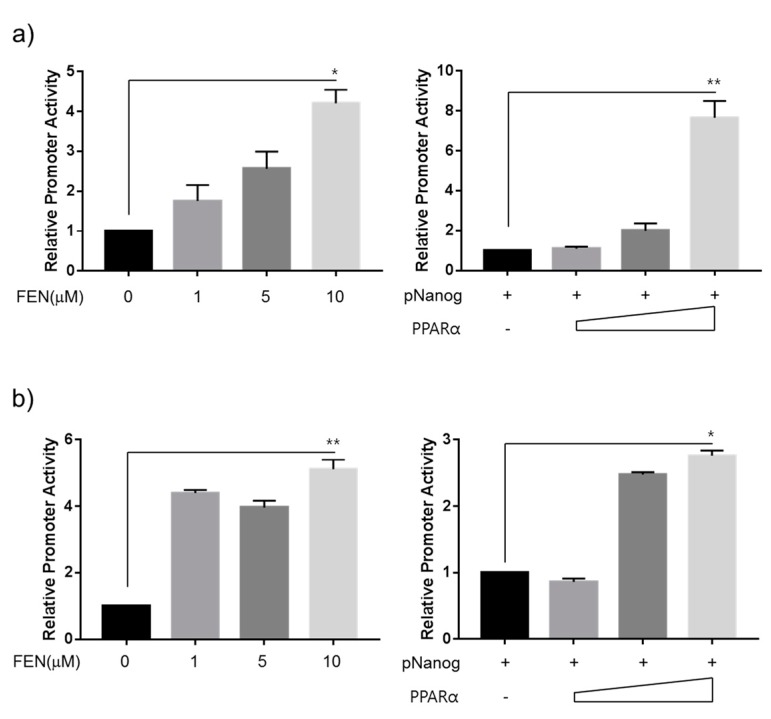
PPARα activates the Nanog promoter. Transcriptional activation of the Nanog promoter by PPARα stimulation in (**a**) J1 ESCs and (**b**) 293T cells. J1 ESCs and 293T cells were transfected with the Nanog5P luciferase reporter plasmid and treated with FEN at the indicated concentrations (a and b, left) the next day. Nanog5P (2 μg) was transfected together with pSG5- PPARα (0, 1, 2, and 4 μg, respectively) into J1 ESCs and 293T cells (a and b, right). Firefly luciferase activity was normalized to renilla luciferase activity to correct for transfection efficiencies.

To examine the binding of PPARα to the Nanog promoter, we performed ChIP assays and qPCR analyses using specific primers to amplify regions of 518 bp that included putative PPRE motifs at −1267 to −1255, −950 to −933, and −840 to −834 bp upstream of the transcription start site (Figure 4). At five days after reprogramming with or without FEN treatment, the reprogrammed cells were fixed, and total chromatin was extracted. The binding of PPARα to the Nanog promoter was observed to be 3-fold enriched in FEN-reprogrammed cells (Figure 4), suggesting that PPARα was specifically recruited by FEN and bound to the Nanog promoter. Next, to determine whether these PPARα binding elements and their binding to PPARα affect Nanog transcription, we performed reporter assays using J1 ESCs and 293T cells transiently transfected with the reporter plasmid Nanog5P, which was constructed by the insertion of 2.5 kb of the 5’ promoter region of the mouse Nanog gene into the pGL3-Basic luciferase vector [33]. Forty-eight h after treatment with FEN, Nanog promoter activity was significantly increased in a dose-dependent manner up to 10 μM in both J1 ESCs (Figure 5a, left) and 293T cells (Figure 5b, left). Similarly, co-transfection with Nanog5P and specific PPARα expression plasmids also increased Nanog promoter activity in both J1 ESCs (Figure 5a, right) and 293T cells (Figure 5b, right), thereby confirming the direct activation of the Nanog promoter by PPARα. These results strongly suggest the existence of active PPARα binding elements within the Nanog promoter region and their functional contribution as a direct target of the PPARα regulatory pathway in the control of pluripotency and reprogramming.

## 4. Discussion

In this study, we unveiled the novel role of PPARα agonists, such as FEN, in the control of the PSC state, especially in the early phase of cellular reprogramming. Moreover, we identified Nanog as a direct target harboring PPRE motifs of PPARα relevant to pluripotency and cellular reprogramming.

Studies have demonstrated the various biological activities of PPARα agonists. Fibric acid derivative (fibrate)-mediated PPARα stimulation increases the gene expression of superoxide dismutase (Sod), glutathione reductase (GR), glutathione peroxidase (GPx), and glutathione-S-transferase (GST), resulting in an enhanced antioxidant and cellular oxidation-reduction (redox) state [40,42]. Fibrates were also found to play an important role in restoring the cellular redox balance in aged mice by regulating oxidative stress via a PPARα-dependent or an independent mechanism [49]. FEN activates nuclear factor erythroid 2-related factor 2 (Nrf2), which is involved in the expression of many antioxidant and detoxifying enzymes by p62-dependent Keap1 degradation [50]. Recent studies have shown that the modulation of PSC fate is partially regulated by reactive oxygen species (ROS), which mediate the redox state of cells as a secondary messenger [51,52,53]. However, thus far, the potential role of PPARα in PSCs has not been elucidated.

**Figure 6:**
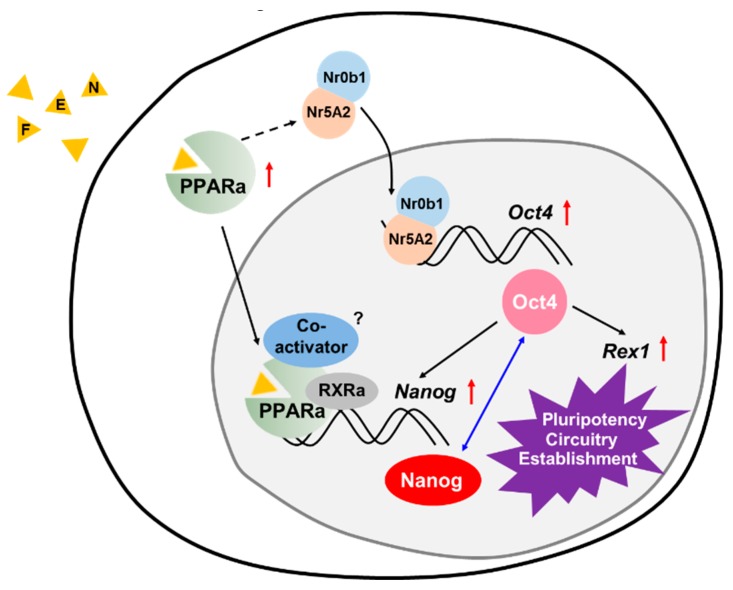
Pharmacological regulation of pluripotency circuitry establishment during cellular reprogramming via the FEN-stimulated PPARα regulatory pathway. Nr5a2 increased by FEN may interact with Nr0b1 and function in Oct4 upregulation together, resulting in the target activation of Oct4 itself, Rex1, and Nanog. Nanog was also identified as a direct target of PPARα and then transcriptionally activated with or without coactivators.

Nr5a2 (also known as LRH-1) was shown to activate the master regulator of stemness Oct4 at the epiblast stage of embryonic development [54] and coactivate Oct4 expression in mouse ESCs by interaction with Nr0b1 (also known as Dax1), which is regarded as an important factor of pluripotency [55], as well as replace Oct4 during cellular reprogramming [13]. Based on these reports, we hypothesized that Nr5A2 increased by FEN may be involved in Oct4 upregulation, resulting in the activation of Oct4 itself, and Rex1 and Nanog, which are well-known Oct4 target genes [56] (Figure 6). However, further investigation is required because PPARα may be involved in the regulation of Oct4 or Rex1, individually and/or directly.

PPARα has been shown to heterodimerize with RXR and recruit spatiotemporally orchestrated associations of coactivators resulting from canonical ligand binding, followed by the ligand-dependent transcriptional activation of target genes involved in diverse biological processes [21,22,23]. These protein complexes can modulate chromatin remodeling, facilitate DNA unwinding by histone acetylation or methylation, and link to the RNA polymerase II machinery for the transactivation of specific target genes [57]. Thus, it may be possible to recruit coactivators or coactivator-associated proteins into the interaction with liganded PPARα within the putative PPARα binding elements of the proximal Nanog promoter, and the unknown sites of the distal Nanog promoter lead to the enhancement of pluripotency circuitry reestablishment (Figure 6). The study of PPARα-specific coactivators for Nanog transactivation will be necessary to further understand the molecular mechanisms underlying PSC fate control.

During PSC induction, the reprogrammed cells have substantially increased ROS levels and oxidative stress [58,59], thereby reducing the survival rate of reprogrammed cells and the generation of iPSCs [60,61]. Interestingly, FEN-mediated PPARα stimulation enhances antioxidant and detoxifying enzymes [50], metabolic shift [62], and the cellular redox state [40,42], suggesting that FEN may modulate PSC fate via a PPARα-dependent or PPARα-independent mechanism. Nanog is also able to quench ROS production and restore OxPhos during metabolic reprogramming, resulting in an enhancement of self-renewal and stemness [43]. According to Nanog ChIP-seq analysis in TICs, Nanog can physically interact with PPARδ and then co-occupy the Acadvl locus, thereby suggesting a possible cooperation to increase FAO in TICs to support self-renewal ability and drug resistance [43]. Thus, PPARα and Nanog may be closely associated with maintaining and reinitiating pluripotency either separately or together. Our results indicate that the positive effects of PPARα agonists on cellular reprogramming in PSC fate by demonstrating PPARα-mediated Nanog regulation may contribute to the beneficial use of FEN in regenerative medicine.

## Supplementary Materials

The following are available online at http://www.mdpi.com/2077-0383/7/12/488/s1, Figure S1: Characterization of FEN-iPSCs, Figure S2: The expression levels of PPARα, Nanog, and Oct4, Figure S3: Homology modeling of PPARα/RXRα. Table S1: A list of compounds tested in this study.
