# Supplementary material for: Peroxisome Proliferator-Activated Receptor α Agonist and Its Target Nanog Cooperate to Induce Pluripotency
Source: J Clin Med. 2018 Nov 27;7(12):488. doi: 10.3390/jcm7120488 (PMC6306698; doi:10.3390/jcm7120488)
Supplement: Supplementary file 1 [file jcm-07-00488-s001.pdf]

Journal of
Clinical Medicine

MDPI

Peroxisome proliferator-activated receptor α agonist and its target Nanog cooperate to induce pluripotency

Jungwoon Lee[1], *Jinhyuk Lee*[2,3], *and Yee Sook Cho*[1,4]

[1]Stem Cell Research Laboratory, Immunotherapy Convergence Research Center, Korea Research Institute of Bioscience and Biotechnology (KRIBB), 125 Gwahak-ro, Yuseong-gu, Daejeon 34141, Republic of Korea

[2]Genome Editing Research Center, Korea Research Institute of Bioscience and Biotechnology (KRIBB), 125 Gwahak-ro, Yuseong-gu, Daejeon, 34141, Republic of Korea

[3]Department of Biotechnology, KRIBB School, University of Science and Technology (UST), 113 Gwahak-ro, Yuseong-gu, Daejeon 34113, Republic of Korea

[4]Department of Bioscience, KRIBB School, University of Science and Technology (UST), 113 Gwahak-ro, Yuseong-gu, Daejeon 34113, Republic of Korea

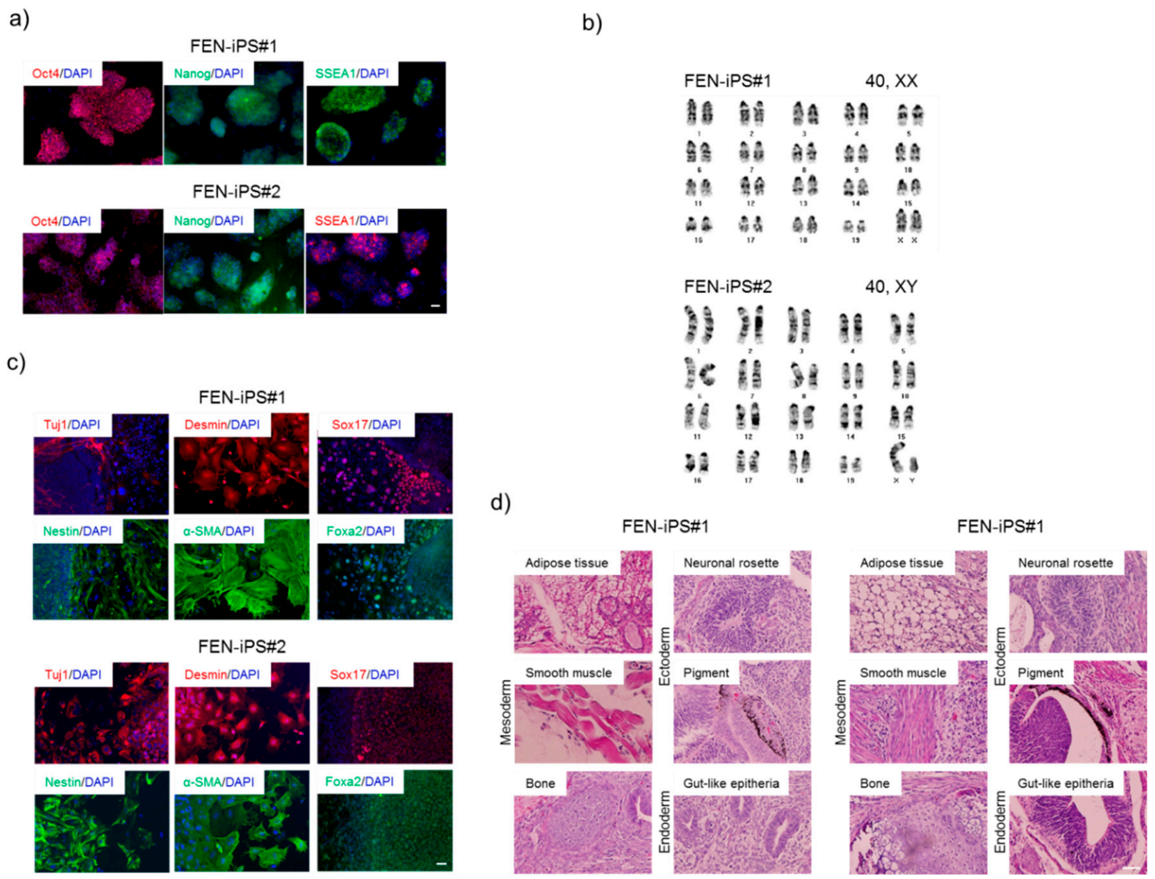

Figure S1. Characterization of FEN-iPSCs. (a) Representative morphology of FEN-iPSC colonies. All iPSC colonies were positive for pluripotency markers (Oct4, Nanog and SSEA1). (scale bar, 20 μm) (b) Normal karyotype of FEN-iPSCs. (c) Immunocytochemistry analysis confirming *in vitro* differentiation into three germ layers. Tuj1 and Nestin for ectodermal markers, Desmin and α-smooth muscle actin (α-SMA) for mesodermal markers, and Foxa2 and Sox17 for endodermal markers. Nuclei were stained with DAPI; blue. (scale bar, 50 μm) (d) *In vivo* three germ layer differentiation by using teratoma formation. Histological analysis by hematoxylin and eosin staining showed that FEN-iPSCs generated representative tissues of ectoderm (pigment and neural rosette), mesoderm (cartilage, adipose tissue, smooth muscle and bone) and endoderm (gut-like epithelia). (scale bar, 50 μm).

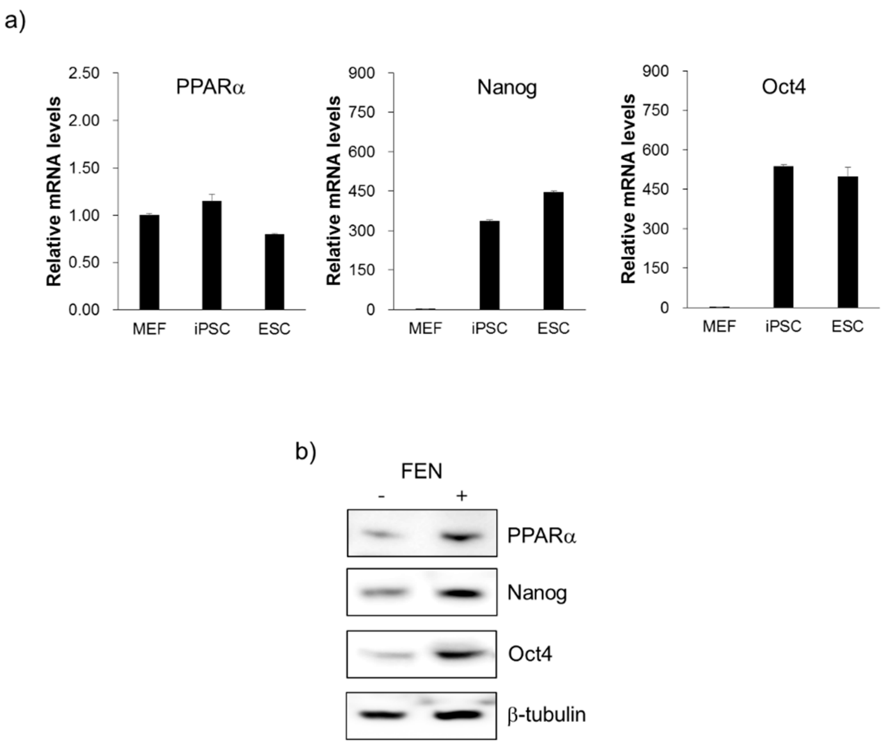

Figure S2. The expression levels of PPARα, Nanog, and Oct4. (a) The gene expression of PPARα, Nanog, and Oct4 in MEFs, FEN-iPSCs, and ESCs. Results were normalized to GAPDH and expressed as fold-increase over MEFs. (b) Western blot analysis shows that FEN stimulates PPARα, Nanog, and Oct4 protein expression. FEN itself increases the expression of PPARα, Nanog, and Oct4 protein in OSKM-induced cells by FEN treatment compared to the control. Protein levels were normalized to β-tubulin for each cell line.

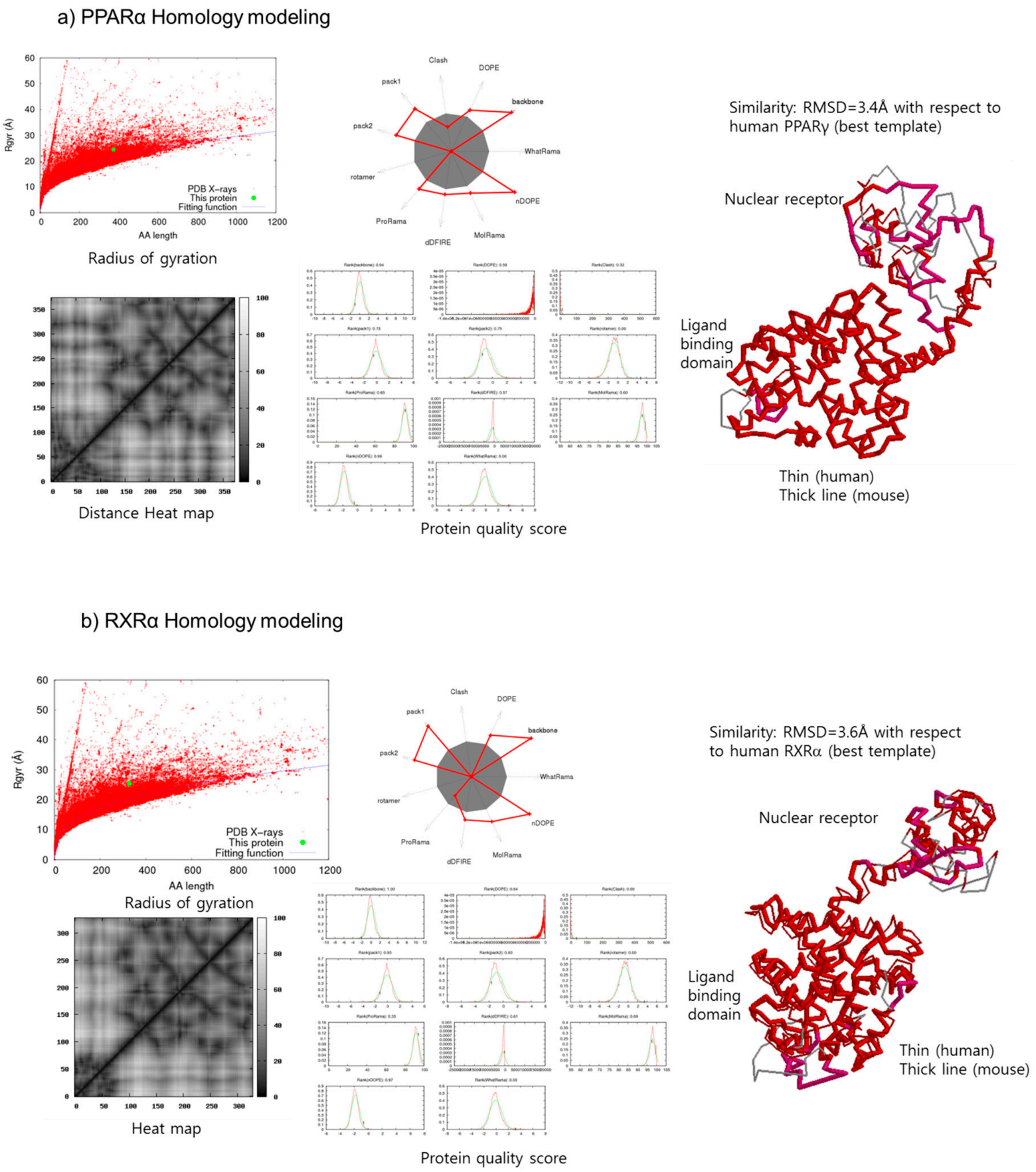

Figure S3. Homology modeling of PPAR*α*/RXR*α*. The generated PPAR*α* and RXR*α* complex is drawn by a trace model (red think line in right subset figure) with a human complex (white thin line). Various quality metrics, such as the radius of gyration, distance heat map, and protein quality score, were measured to validate whether the structure was good.
